# Determining Conditions for Successful Culture of Multi-Cellular 3D Tumour Spheroids to Investigate the Effect of Mesenchymal Stem Cells on Breast Cancer Cell Invasiveness

**DOI:** 10.3390/bioengineering6040101

**Published:** 2019-11-01

**Authors:** Marie-Juliet Brown, Soukaina Bahsoun, Mhairi A. Morris, Elizabeth C. Akam

**Affiliations:** School of Sport, Exercise and Health Sciences, Loughborough University, Loughborough LE11 5DU, UK; M.J.Brown2@lboro.ac.uk (M.-J.B.); S.Bahsoun@lboro.ac.uk (S.B.); M.A.Morris@lboro.ac.uk (M.A.M.)

**Keywords:** mesenchymal stem cells, breast cancer cells, tumour spheroids, invasion

## Abstract

Mesenchymal stem cells have been widely implicated in tumour development and metastases. Moving from the use of two-dimensional (2D) models to three-dimensional (3D) to investigate this relationship is critical to facilitate more applicable and relevant research on the tumour microenvironment. We investigated the effects of altering glucose concentration and the source of foetal bovine serum (FBS) on the growth of two breast cancer cell lines (T47D and MDA-MB-231) and human bone marrow-derived mesenchymal stem cells (hBM-MSCs) to determine successful conditions to enable their co-culture in 3D tumour spheroid models. Subsequently, these 3D multi-cellular tumour spheroids were used to investigate the effect of hBM-MSCs on breast cancer cell invasiveness. Findings presented herein show that serum source had a statistically significant effect on two thirds of the growth parameters measured across all three cell lines, whereas glucose only had a statistically significant effect on 6%. It was determined that the optimum growth media composition for the co-culture of 3D hBM-MSCs and breast cancer cell line spheroids was 1 g/L glucose DMEM supplemented with 10% FBS from source A. Subsequent results demonstrated that co-culture of hBM-MSCs and MDA-MB-231 cells dramatically reduced invasiveness of both cell lines (F_(1,4)_ = 71.465, *p* = 0.001) when embedded into a matrix comprising of growth-factor reduced base membrane extract (BME) and collagen.

## 1. Introduction

Human mesenchymal stem cells (hMSCs) are multi-potent, non-haematopoietic cells that are plastic-adherent and can self-renew with the ability to differentiate into multiple mesenchymal lineages (osteoblasts, adipocytes, and chondrocytes). These cells can be readily isolated and expanded in vitro, originally and most commonly from bone marrow (hBM-MSCs), but other sources of hMSCs have since been identified, including adipose tissue, peripheral blood, and umbilical cord blood [1]. Recently, hMSCs have been implicated in tumour growth and progression in several ways. They have been found to play a role in the promotion of the epithelial to mesenchymal transition (EMT), a key biological process that can promote metastasis and alter the invasiveness and aggressiveness of cancer cells [2]. hMSCs can interact with tumour cells, directly and indirectly, via cell–cell interactions and paracrine signalling, resulting in altered proliferation, metabolic activity, and mobility of breast cancer cells [3,4,5,6]. They may also play a critical role in extracellular matrix (ECM) remodelling, again altering the invasive tendencies of cancer cells [7]. However, there are discrepancies on the impact of hMSCs on tumour progression, with an almost equivalent volume of literature demonstrating anti-tumourigenic effects of hMSCs. Studies have shown that hMSCs can inhibit angiogenesis and stimulate apoptosis [8], and several reviews of the topic effectively collate the evidence for the tumour suppressive effects of hMSCs [2,9,10]. 

T47Ds are a non-invasive breast cancer cell line under the luminal A classification; low grade, often slow growing cancers that start in the inner (luminal) cells of the mammary duct and have good prognosis [11]. Generally, these cancer types are oestrogen receptor α-positive and human epidermal growth factor receptor (HER2)-negative. Luminal A cancers are the most common molecular subtype of breast cancers [12]. MDA-MB-231 cells are highly invasive cancer cells; they are oestrogen receptor α-negative, progesterone receptor-negative, and HER2-negative, and can be classified as basal B or triple negative cancer cells. These cells have a mesenchymal phenotype as a result of undergoing EMT, rendering them with a long, spindle-shape form and extremely invasive tendencies. Almost 90% of cancer-related deaths result from cancer cell metastasis [13], and therefore, investigating the role of hBM-MSCs on tumour progression and metastasis is critical.

In vitro mammalian tissue culture procedures are often conducted following local practice. As a consequence, suboptimal procedures and techniques may be used and cell culture-associated problems are ignored [14]. In vitro tissue culture methods use culture media to maintain and support mammalian cell growth. Basal culture media is produced to meet the generalised nutritional and environmental requirements of mammalian cell lines, typically containing components such as vitamins, salts, amino acids, and glucose. This mix of components is highly suited for the growth of a broad spectrum of mammalian cells by providing an appropriate source of energy and compounds which regulate the cell cycle. 

Culture media is often supplemented with serum, such as foetal bovine serum (FBS), and other components to create complete media that can support survival, proliferation, and function of cells. FBS works by providing a broad spectrum of proteins such as hormones and growth factors, carriers, or chelators for labile or water-insoluble nutrients. However, supplementing media with serum or serum-derived components can cause variation in cell morphology, proliferation, and chemotactic responses [15]. Different sera comprise of a large number of growth-mediating constituents, some that are often undefined and/or unquantified, and as well as positive growth-mediators, sera can also include contaminants and viruses which may negatively regulate growth [15]. Despite moves to stop the use of FBS in cell culture in line with scientific, safety, and ethical grounds, animal-derived serum remains an extremely common feature of cell culture methods due to its lower cost and ease of availability. 

More recently within in vitro mammalian tissue culture, the importance of the development and drive towards using three-dimensional (3D) models to analyse tumours and the therapeutic potential of hMSCs is becoming recognised. A major limitation of two-dimensional (2D) cell culture is the insufficient cell–cell cross talk. In vivo, cells grow close together in a 3D environment, and therefore, in a 2D culture platform, important cell–cell interactions may be missed. Evidence that properties of cancer cells and stem cells cultured in 3D environments are significantly different to those cultured in 2D environments is accumulating, and it is important that the focus for future research is on 3D models, to reduce differences between in vivo and in vitro research environments.

This study focused on altering serum source and glucose concentrations to determine successful co-culture conditions for two breast cancer cell lines (T47D and MDA-MB-231) with human bone marrow-derived mesenchymal stem cells (hBM-MSCs), such that the interactions between these cell types could be assessed using 3D tumour spheroids. Of significant interest was the variability on growth caused by FBS from different sources, together with the elucidation of the most appropriate glucose concentration for all three cell lines. The establishment of validated and optimised co-culture conditions enabled 3D heterotypic and homotypic tumour spheroids to be successfully cultured. This then facilitated the assessment of the invasion of T47D and MDA-MB-231 cells when co-cultured with hBM-MSCs in a 3D tumour spheroid model, enabling the type and direction of the interaction to be analysed.

## 2. Materials and Methods 

Initially, cellular growth curves were conducted to establish the effects of glucose concentration and source of serum on different cell lines to determine the best culture conditions for prospective 3D tumour co-culturing of these cells. The following cell lines were used: T47D (human, ductal carcinoma of the breast; ECACC 85102201) and MDA-MB-231 (human Caucasian breast adenocarcinoma, invasive; ECACC 92020424) cell lines supplied by the European Collection of Authenticated Cell Cultures, and normal human bone marrow-derived mesenchymal stem cells (hBM-MSCs) were supplied by Lonza Poietics™, Mapleton, IL, USA (Catalogue number: PT-2501; Lot number: 071313B).

### 2.1. Glucose and Serum Conditions

In order to establish whether there is a significant effect of glucose concentration and/or source of sera on cell growth, cells were cultured in Dulbecco’s Modified Eagle Medium (DMEM; Gibco™, Thermo Fisher, Loughborough, UK) low glucose (1 g/L) or with DMEM high glucose (4.5 g/L; Gibco™, Thermo Fisher UK), both supplemented with GlutaMAX (3.97 mM; Thermo Fisher, Loughborough, UK) and pyruvate (1 mM, ThermoFisher, UK). Media was completed with 10% foetal bovine serum (FBS) from one of two different sources/suppliers, designated A and B, both serum sources had a comparable source of origin (USA; please see Appendix B for supplier information). 

In total, each cell line was cultured in four different growth medium compositions across a period of 7 days. Each of the four variants of growth media were designated as follows:

DMEM low glucose (1 g/L) supplemented with 10% FBS from source A (LG-A).

DMEM high glucose (4.5 g/L) supplemented with 10% FBS from source A (HG-A).

DMEM low glucose (1 g/L) supplemented with 10% FBS from source B (LG-B).

DMEM high glucose (4.5 g/L) supplemented with 10% FBS from source B (HG-B).

### 2.2. Growth Curves

Cryopreserved human mesenchymal stem cells (P5), T47D (P4) cells, and MDA-MB-231 (P4) cells were recovered and cultured in Corning® T75 flasks (Sigma-Aldrich, St. Louis, MO, USA) for a period of 5–7 days to allow for recovery from cryopreservation and for confluency to be reached. Upon reaching 70–90% confluency, cell numbers were counted, and this information was used to accurately seed 7 Corning® T25 flasks per condition (four conditions) and for each cell line (three cell lines) at a density of 5000 cells/cm^2^. Cells were then cultured for 7 days in a humidity chamber at 37.5 °C, 5% CO_2_, and cell numbers were counted at equal intervals (approx. 24 h) each day. Media were replaced every three days. Each growth curve was repeated three times to obtain triplicate results.

### 2.3. Daily Cell Counting Protocol

At approximately 24 h intervals, one flask from each condition was counted using the Countess™ automated cell counter (Invitrogen, Thermo Fisher, Loughborough, UK). Spent media were removed and the cell monolayer was washed twice. First with 1× PBS (Fisher Scientific, UK), followed by PBS-EDTA (5 µM; Invitrogen, UK) to reduce clumping of cells. After washing, 2 mL of Trypsin (Fisher Scientific, UK) was added and incubated at 37 °C for up to 5 minutes to allow cells to detach. Then, 4 mL of the appropriate growth media was then added to inactivate the trypsin. The cell suspension was centrifuged at 200× *g* for 5 minutes at 21 °C. The resulting cell pellet was re-suspended in 1 mL of the appropriate media. A volume of the cell suspension was mixed with an equal volume of trypan blue stain. Next, 10 µL of this cell-stain mixture was added to each chamber of a Countess™ cell counting slide and counts of the total number of cells, number of live cells, dead cells, and viability counts were obtained for each flask. Specific growth rate (SGR), population doubling level (PDL), population doubling time (PDT), and fold increase (FI) were calculated using N_0_ (seeding density) and N_x_ as the final number of cells on day 7 (see Appendix A for calculations).

### 2.4. hBM-MSC Immunophenotyping

Surface marker expression of hBM-MSCs cultured in source A serum was analysed by flow cytometry using an MSC (human) phenotyping kit (Miltenyi Biotec, Bisley, UK) according to manufacturer’s instructions. To confirm compliance with the International Society for Cell and Gene Therapy (ISCT) minimum criteria for defining hBM-MSCs [16], positive markers stained for were CD105 linked to PE, CD90 linked to FITC, and CD73 linked to APC. Again, to fully comply with ISCT minimum criteria, negative markers also stained for included CD14, CD20, CD34, CD45, and HLA-DR, which were all linked to PerCP. In brief, approximately 5 × 10^5^ cells were suspended in 100 µL of flow cytometry buffer. Then, 10 µL of hMSC phenotyping cocktail and 10µL of Human Anti-HLA-DR-PerCP were added and mixed. Cells were then incubated in the dark for 10 minutes at 5 °C. Then, cells were washed with buffer and subsequently centrifuged prior to re-suspension in 500 µL of fresh buffer for analysis. Unstained samples and corresponding isotype controls were also prepared and analysed for control purposes. The BD Accuri C6 was used for analysis, with a minimum of 100,000 events collated for each sample, and the resulting data were then analysed using BD Accuri C6 plus software.

### 2.5. Fluorescent Staining of Cells for Spheroid Formation

Cells that had reached 70–90% confluence were stained using the following CellTracker™ fluorescent probes (ThermoFisher Scientific, UK): CellTracker™ Green CMFDA, CellTracker™ Orange CMRA, and Cell Tracker™ Deep Red. Cells were stained following the manufacturer’s instructions. Briefly, anhydrous dimethyl sulfoxide (DMSO) was added to the lyophilised product to create 10 mM stock solutions of Green CMFDA and Orange CMRA dyes, and 1 mM stock solutions of the Deep Red tracker dye. Next, 20 µM working solutions of the Green and Orange dyes were obtained by adding the appropriate volume of stock solution to the specific growth medium. Due to the high fluorescent signal obtained from the Deep Red dye, the working concentration used was 1 µM. 

Cells in culture flasks had media removed and were incubated at 37 °C/5% CO_2_/95% humidity with the dyes for 30–45 minutes. The CellTracker™ working solutions were then removed, and cells were washed with 5 mL 1× PBS twice, before continuing appropriate experimental procedures.

### 2.6. PDMS Coating

In order to encourage spheroid formation within a shorter time period, spheroids were cultured using 60 mm dishes coated with polydimethylsiloxane (PDMS) elastomer. The SYLGARD 184 Silicone Elastomer Kit (Dow Corning, Midland, MI, USA) was used. A silicone elastomer base was combined with a curing agent at a ratio of 10:1 (according to manufacturer’s instructions) to form the PDMS elastomer. This was then carefully and evenly poured directly into 60 mm dishes. Following this, dishes were either cured over night at room temperature, or heat cured at 50 °C for approximately 4–5 h. Finally, culture dishes were re-sterilised under UV light in a laminar flow hood before use. 

### 2.7. Spheroid Formation

Adherent cell cultures of T47D, MDA-MB-231, and hBM-MSCs were grown to 70–90% confluence in T75 flasks. Cells were then stained using the previously mentioned protocol (Section 2.5), if required. The cells were washed twice with 1× PBS, followed by detachment from flasks by incubating with 4 mL of TrypLE enzyme dissociation solution (Thermo Fisher Scientific, UK) for 5 minutes at 37 °C. TrypLE was deactivated by adding 4 mL of defined growth media, and this cell suspension was transferred to 15 mL centrifuge tubes. Cell suspensions were centrifuged at 200× *g* for 5 minutes at 21 °C. Supernatant was discarded, and cell pellets were resuspended in variable volumes of media, depending on pellet size, to allow for accurate quantification of the cell numbers by cell counting (refer Section 2.3). 

The correct volume of this cell suspension was then taken to obtain a cell suspension with a concentration of 3 × 10^5^ cells/mL, to seed 4500 cells per 15 µL hanging drop. The lid of a PDMS-coated 60 mm tissue culture dish was removed, and 5 mL 1× PBS was added to the lid, creating a hydration chamber for cell growth in hanging drops. The base was inverted and around 35 × 15 µL drops were placed onto the dish. Keeping the dish inverted and the lid on the bottom, the dish was incubated at 37 °C/5% CO_2_/95% humidity, for 24–48 h to allow spheroid formation to occur. If cells required more than 48 h to form spheroids, around 8–10 µL of media from the drop was removed and replaced with fresh media. Formation was checked daily using an inverted microscope.

### 2.8. Embedding Spheroids

Single spheroids were embedded into a matrix consisting of growth-factor reduced base membrane extract (BME; 8–12 mg/mL; Bio-Techne Ltd., Abingdon, UK) and rat tail collagen type 1 at 5 mg/mL (R&D Systems, Minnneapolis, MN, USA). BME was thawed on ice, along with rat collagen type I, and 10 µL of each (1:1 ratio) were collected into pre-chilled 0.5 mL tubes. Then, 10 µL of the hanging drop suspension, including the spheroid, was collected using 20 µL pipette tips that were cut to widen the tip and avoid spheroid disruption. Immediately, this was added to the BME + collagen mixture. The 30 µL solution was then placed into a single well of a 24-well plate. This procedure was repeated to obtain the triplicates of spheroid containing wells. Spheroids were left for 5 days embedded in the matrix with media replaced on the third day. Images were taken daily.

### 2.9. Imaging of Spheroids

Spheroid formation in hanging drops was observed by imaging daily using an inverted laboratory Leica microscope, a 4× or 10× objective, with attached Leica MC170 HD camera, and Leica Application Suite (LAS) software (Leica Microsystems, Milton Keynes, UK). Fluorescent images of embedded spheroids were obtained using the LEICA DM IL LED with the LEICA DFC360 FX 1.4 mega-pixel camera, a 4× or 10× objective, and the LAS X software. For each spheroid, lighting and focus were adjusted to provide maximal contrast between the 3D structure and background. The emission and excitation filters used for the CellTracker™ dyes are shown in Table 1. 

### 2.10. Analysis of Invasion 

Invasion was calculated using the multi-point tool on ImageJ. The co-ordinates of the cellular core centre were measured and then co-ordinates for the outer most invaded cells (perimeter of invaded area) were collected. The linear distance of each invaded cell from the centre of the core was then calculated. In order to calculate spheroid size across time, images were inverted using ImageJ, converted to an 8-bit type image, and thresholds were adjusted to allow for accurate automatic particle analysis. 

### 2.11. Statistical Analysis

All statistical analyses were run using IBM SPSS Statistics 23 (IBM Corporation, New York, NY, USA). Initially, data were analysed to assess normality and homogeneity of variances. Normally, distributed data were analysed using one-way between-measures analysis of variances (ANOVAs). In the case where the data did not meet parametric assumptions, data were transformed (log transformation), and parametric tests were then conducted. *p* < 0.05 was considered significant. 

## 3. Results

### 3.1. Growth Curves

#### 3.1.1. hBM-MSCs

**Figure 1:**
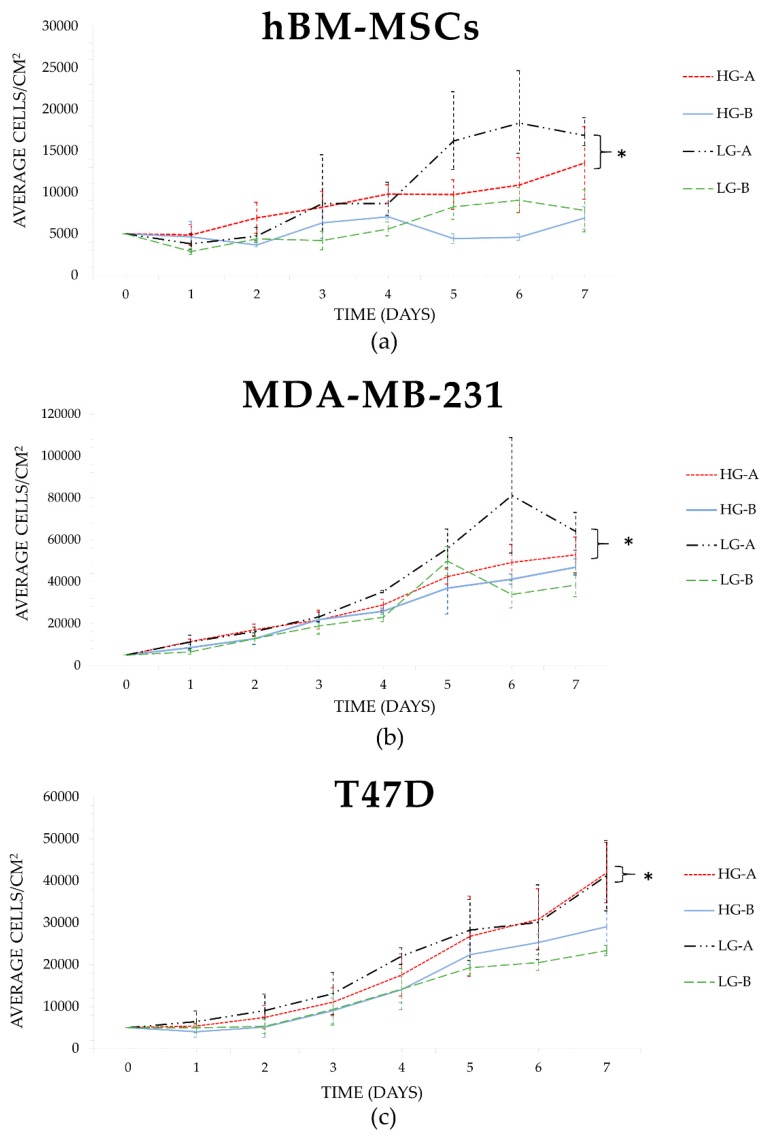
Cellular growth curves of hBM-MSCs, MDA-MB-231, and T47D cells under four different growth media combinations. Significance depicted by *. (**a**) hBM-MSCs showed a significant difference between cell numbers on day 7 in serum source A and B (*p* = 0.002). (**b**) MDA-MB-231 growth curve shows highest growth in LG-DMEM supplemented with serum A. Changes in glucose concentration had a significant effect on average day 7 cell numbers (*p* = 0.005). (**c**) T47D growth curve indicating a significant difference between growth in serum A vs B (*p* ≤ 0.042), but no significant preference for LG or HG DMEM when complete with FBS from source A (*p* > 0.05).

Clear differences in growth were observed across the different growth media compositions between the three cell lines tested. Glucose concentration changes appeared to have relatively little effect on hBM-MSCs growth, where LG-A and HG-A conditions performed similarly across all growth parameters, as did LG-B and HG-B conditions. However, there were clear differences in growth between the two serum sources; the two growth media conditions supplemented with source A serum had noticeably greater cell growth than the source B conditions (refer to Figure 1). Population doubling times averaged 2.68 days in serum A, compared to an average population doubling time of 8.77 days in serum B. Statistical analyses of the growth parameters revealed that serum source had a statistically significant effect on hBM-MSC cell numbers at day 7 (*p* = 0.002), but glucose concentration did not. For hBM-MSCs, it was apparent that LG-DMEM supplemented with 10% FBS from source A was the optimum media composition out of the four tested; however, the HG-A condition was not significantly different. Of note, hBM-MSCs underwent immunophenotyping and data from flow cytometry revealed that hBM-MSCs used in this study met the minimum defined International Society for Cell and Gene Therapy (ISCT) surface marker expression criteria, as more than 95% of the population were positive for CD105, CD90, and CD73, and less than 0.1% of the population were negative for CD14, CD20, CD34, CD45, and HLA-DR (see Supplementary Figure S1).

#### 3.1.2. MDA-MB-231s

The growth of MDA-MB-231s appeared to have a greater response to different media compositions when compared to hBM-MSCs and T47Ds. LG-A conditions led to the greatest proliferation of MDA-MB-231 cells compared to the other conditions across the growth period, with a total day 7 mean of 64,000 cells/cm^2^ compared to 52,800, 47,000, and 38,400 cells/cm^2^ for HG-A, LG-B, and HG-B conditions, respectively (refer to Figure 1). Average population doubling time was 1.10 days across source A conditions, and 1.84 days in source B conditions. Statistical analyses of growth parameters showed that serum had a statistically significant effect on almost all growth parameters (*p* ≤ 0.049). Changes in glucose concentration had a significant effect on average day 7 cell numbers (*p* = 0.005), but not on any other growth parameters (*p* > 0.05; see Table 2), which suggests that serum source has the greatest impact on growth compared to glucose concentration. The overall ‘optimal’ growth media composition for MDA-MB-231 cells out of the four combinations tested appeared to be low glucose DMEM supplemented with 10% FBS from source A, which was consistent with the hBM-MSCs’ preferred composition.

#### 3.1.3. T47Ds

T47Ds also displayed a preference for source A serum over source B serum, where cells cultured in both low glucose and high glucose DMEM supplemented with source A serum exhibited greater growth increases than both source B supplemented conditions (see Figure 1). Interestingly, it appeared that HG-A was the growth media composition that resulted in the best growth parameter averages (day 7 cell numbers, population doubling level, population doubling time, and fold increase) over LG-A conditions (see Table 2). Growth was consistently lowest when T47Ds were cultured in LG-B growth media. Statistical analyses revealed that the difference in growth as a result of serum changes was statistically significant (*p* ≤ 0.042), whereas glucose concentration did not have a statistically significant effect on growth (*p* > 0.05), indicating serum source choice is potentially a more important factor for the growth of T47D cells.

### 3.2. Effect of Co-culture on Invasion of Breast Cancer Cells (BCCs) and hBM-MSCs

**Figure 2:**
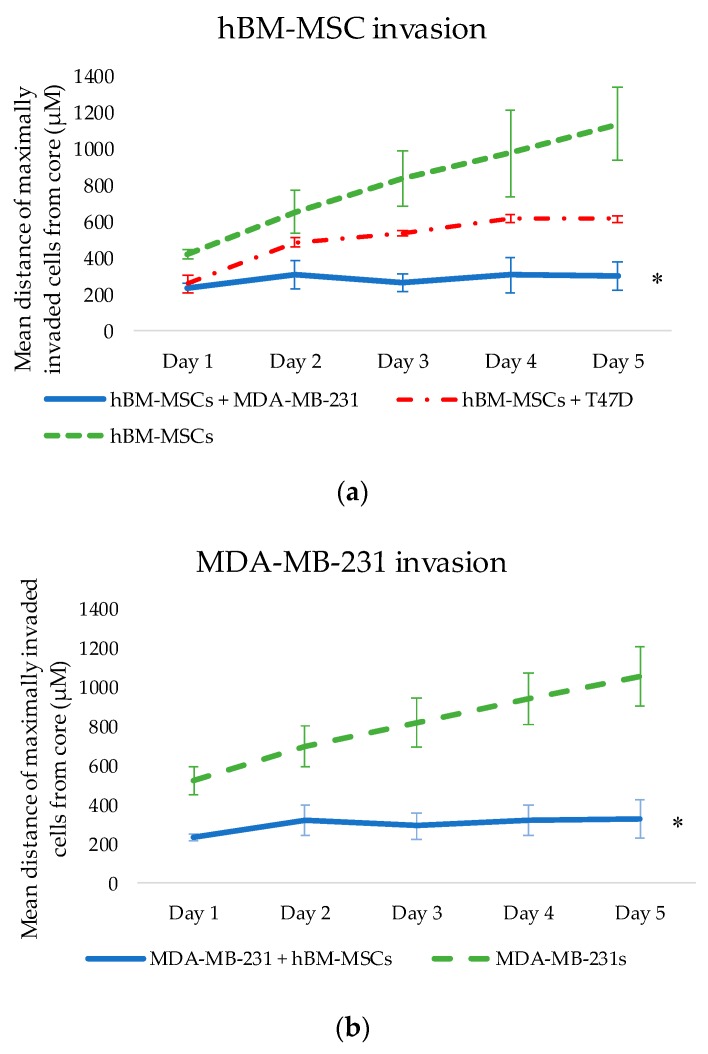
(**a**) Invasion of hBM-MSCs in homotypic spheroids or heterotypic spheroids (hBM-MSCs + MDA-MB-231 or hBM-MSCs + T47D) across a 5-day period. Significance depicted by *. hBM-MSCs in 3D spheroids have a greater invasive capacity when cultured alone, compared to when cultured with breast cancer cells (*p* < 0.05). Culturing hBM-MSCs with MDA-MB-231 cells significantly reduced hBM-MSC invasion. (**b**) Invasion of MDA-MB-231s in homotypic or heterotypic spheroids (MDA-MB-231s + hBM-MSCs). MDA-MB-231 invasion reduced significantly when in a co-cultured spheroid with hBM-MSCs compared to invasion of MDA-MB-231s in a homotypic spheroid (*p* = 0.001).

From co-culture, our results show that there were significant differences between the invasion of homotypic hBM-MSC spheroids (hBM-MSCs alone) and heterotypic spheroids (hBM-MSCs + MDA-MB-231s, or hBM-MSCs + T47Ds). A significant main effect for condition (hBM-MSCs alone, +MDA-MB-231s, or +T47Ds; F_(2,6)_ = 108.208, *p* < 0.005) revealed that hBM-MSC invasion was lowest when cultured with MDA-MB-231s, compared to when cultured alone or with T47Ds. When cultured as single-cell-type 3D spheroids, hBM-MSCs demonstrated high invasiveness across the 5-day period; the average maximal invasive distance for hBM-MSC spheroids was 1137 ± 200.3 µM from the core on day 5. In comparison, co-culture of hBM-MSCs with MDA-MB-231 cells or T47D cells in a 3D tumour spheroid appeared to decrease invasion of hBM-MSCs, where the maximal invasive distance on day 5 was 303 ± 77 µM and 616 ± 16.71 µM, respectively. Similarly, results show that there was a significant effect of condition (MDA-MB-231s alone or + hBM-MSCs; F_(1,4)_ = 71.465, *p* = 0.001), where invasion of MDA-MB-231s cultured in tumour spheroids with hBM-MSCs was significantly lower at all time points (see Figure 2b), compared to the control.

**Figure 3:**
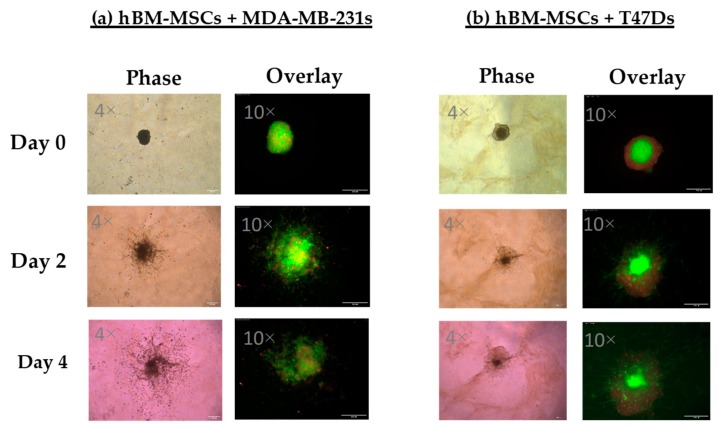
Representative images showing invasion of multi-cellular tumour spheroids consisting of MDA-MB-231 (**a**) or T47D (**b**) cells combined with hBM-MSCs in a 1:1 ratio, when embedded into a collagen:BME matrix.

**Figure 4:**
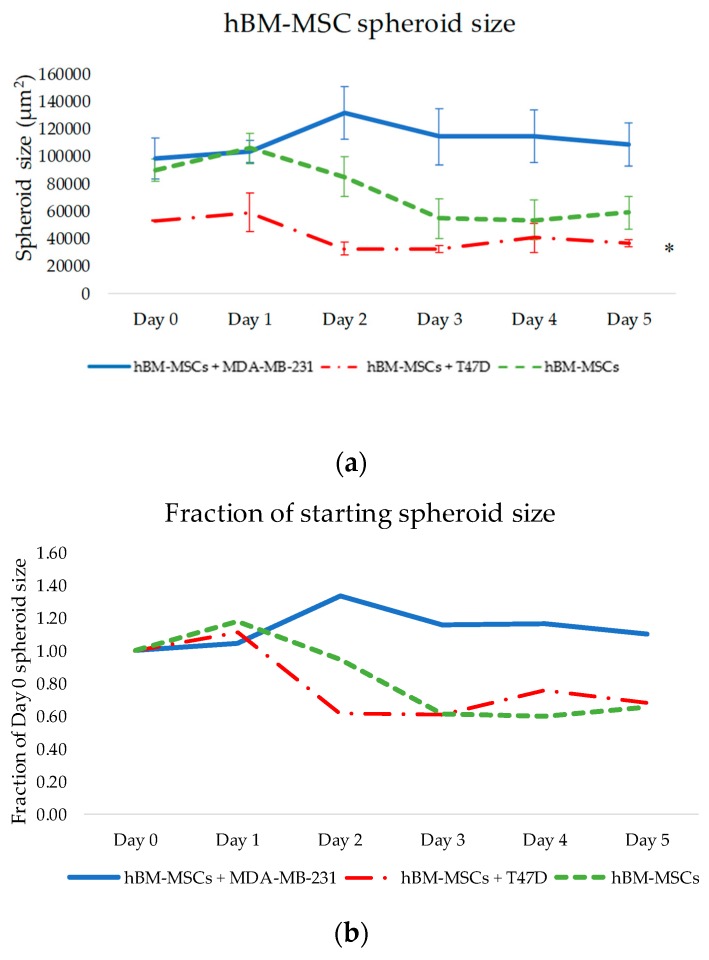

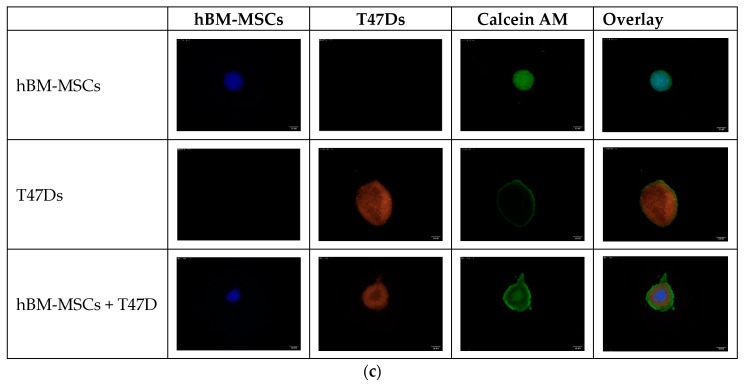
(**a**) Change in spheroid size of heterotypic hBM-MSCs + cancer cell spheroids and homotypic hBM-MSC spheroids across a period of 5 days. Significance depicted by *. Combining hBM-MSCs with T47D cells led to formation of the smallest hBM-MSCs spheroids, compared to hBM-MSCs with MDA-MB-231, which formed large, less compacted spheroids (*p* = 0.001). (**b**) Size of spheroids in co-culture across 5 days as a fraction of starting size on day 0. (**c**) Representative images of hBM-MSC spheroid size when cultured as spheroids alone, and with T47D cells at a 1:1 ratio. hBM-MSCs formed a small, compact core spheroid in the middle of the T47D + hBM-MSCs spheroid. hBM-MSCs and T47Ds were fluorescently stained with CellTracker™ dyes Deep Red (blue filter applied on ImageJ) and Orange CMRA, respectively. Images taken at 4× magnification; scale bar is 250 µM.

A significant effect of the co-culture conditions revealed that culture of hBM-MSCs in tumour spheroids with MDA-MB-231s and T47Ds had a highly significant effect on the spheroid size of hBM-MSCs across 5 days (hBM-MSCs alone, +MDA-MB-231s or +T47Ds; F_(2,6)_ = 357.792, *p* = 0.001). Figure 3 shows representative images of MDA-MB-231 or T47D spheroids combined with hBM-MSCs. Results show that hBM-MSC spheroid size was smallest when cultured with T47D cells (see Figure 4a). Figure 4b presents spheroid size as a fraction of its starting size using average spheroid size values (size on day 0).

## 4. Discussion

In this study, the effect of glucose concentration and FBS source on cell growth and the effect of co-culturing hBM-MSCs and breast cancer cells in 3D tumour spheroid models were investigated. It was anticipated that glucose concentration would have the greatest influence on cell growth, since glucose plays such a substantial role in growth and survival, and when purchasing culture medium, glucose concentration can be specifically selected. However, these results show that serum source presented the greatest changes in cell growth in this study. Of the 15 measurements of growth taken across the three cell lines, serum source had a statistically significant effect on two-thirds of them, whereas glucose concentration only statistically significantly affected 6%. MDA-MB-231 cells exhibited shorter average population doubling time and a greater average fold increase in low glucose and high glucose conditions supplemented with source A serum, compared to source B (1.01 days and 11.68 compared to 1.25 days and 8.54). Nearly all growth measurements for T47D cells and MDA-MB-231s were significantly affected by serum source (Table 1), whereas hBM-MSCs appeared to be less sensitive to changes in serum and glucose conditions.

To further our understanding, the certificates of analysis from both batches of serum were compared to distinguish any key chemical compositional differences between sources to evaluate potential reasons behind the differential growth effects. The chemical compositions of both sera held obvious disparities (refer to Table 3). 

Serum albumin, the most abundant protein in human serum, has been well documented as having inhibitory effects on cancer cell growth. A meta-analysis summarising evidence from epidemiological literature on the association between serum albumin levels and survival in several cancer types found that lower serum albumin levels were associated with poor patient survival in female cancers [17], lending further credence to the observed inhibition of cancer cell growth. Consequently, this evidence therefore aligns with results from this present study, since cancer cell growth in source B serum (with almost 3-fold greater levels of albumin compared to source A) was significantly lower in both cancer cell lines. hBM-MSCs also displayed lower growth in source B conditions compared to A; however, limited literature exists on the effect of albumin on hMSC growth for comparison. Therefore, future studies may reveal the influence of albumin on hMSC growth too.

Another key difference between serum sources was the total cholesterol levels, and evidence suggests the possible involvement of cholesterol pathways in cancer [18] through various mechanisms. Cholesterol can be catabolised into 27-hydroxycholesterol (27-HC), which can act on oestrogen receptors to stimulate breast cancer tumour growth, and liver X receptors to influence EMT and metastasis [19]. Both cancer cell lines had greater levels of growth in the serum with lower cholesterol levels in the present study. Future studies could include invasion assays where levels of cholesterol are altered manually to investigate more thoroughly the effect of cholesterol levels on in vitro growth of T47D and MDA-MB-231 cancer cells lines. Literature also suggests that high cholesterol levels can alter hMSC differentiation preferences [20]. Conducting further research on the effects of serum source and/or cholesterol differences on differentiation preferences of hMSCs could be of value, especially within regenerative medicine.

Another variable that differed in concentration between serum source was haemoglobin. As both our cancer cells and hBM-MSCs demonstrated greater growth in serum A conditions, it is important to understand whether serum haemoglobin concentrations could have contributed to this. Hypoxic environments are already a common pathophysiological feature of tumour microenvironments and advanced cancers, due to the vast demands of rapidly dividing cells and insufficient or ineffective vascular supplies [21]. Hypoxic conditions lead to the release of hypoxia-inducible factors (HIFs), which can contribute to the process of increased vascularisation by targeting genes involved in angiogenesis, generating favourable conditions for cancer cell proliferation, survival, and metastases [22]. Similar to cancer cells, it has been found that hMSC proliferation, migratory capacity, and function can be enhanced in hypoxic conditions. It is reported that low O_2_ percentages (3%) can stimulate a greater yield of hMSCs when cells are cultured in low oxygen for a number of days [23], and can significantly enhance proliferative ability by approximately 10 population doublings compared to ambient O_2_ levels (20%) [24]. Therefore, from the wider literature, it can be seen that both cancer cells and hMSCs can thrive in hypoxic conditions in vitro, where other mammalian cells would perish. It could be speculated that serum with low levels of haemoglobin could have contributed to increased proliferation compared to serum with higher haemoglobin levels (source B serum). Conversely, reported haemoglobin levels may simply be an indication of serum quality to reflect upon the cell fractionation and production process of the serum, allowing for relative homogeneity between batches to be checked and controlled. Again, further research investigating in more detail the effect of varying levels of haemoglobin and/or serum purity on the co-culture of these cells is required. 

The concentration of immunoglobulin (Ig) G in source B serum was 598 µg/mL, compared to 81 µg/mL in source A serum. This is just over a 7-fold difference, and one of the more significant differences in composition of the serum sources tested. Cancer is immune evading and it is speculated that cancer prefers growth in conditions with low immunoglobulin presence. The functional role of immunoglobulins has been investigated using anti-human IgG in mice, where it was found that tumour volume reduced in animal models with added IgG and anti-human IgG induced apoptosis in cancer cell culture [25]. It has also been demonstrated that immunoglobulins can be produced and expressed independently by cancer cells of epithelial origin. Studies suggest that surface-expressed Igs from cancer cells (cIgGs) may have the opposite effect to B lymphocyte-produced IgGs, and may instead be essential for cancer growth by interacting with serum components that are harmful to cancer cells [25]. In this study, growth was more successful in conditions with serum A, containing much lower levels of IgG compared to serum B. Assuming the IgG present in the serum was not produced or expressed by cancer cells, these findings support the anti-tumourigenic role of IgG.

In order to investigate thoroughly the impact of hBM-MSCs on MDA-MB-231 and T47D breast cancer cell lines, two commonly used cell lines in tumour research, this initial research was critical. Choosing the most appropriate cell culture methods will allow us to better understand and manipulate the tumour microenvironment in vitro for all future research on this topic. Moreover, the findings presented herein highlight the importance of testing the impact of different serum sources on the growth characteristics of individual cell lines, and not assuming that all serum sources are equal.

### Co-Culture of Cancer Cells with hMSC

From the thorough and effective selection of a compatible growth media, single-cell type and co-cultured 3D tumour spheroids (cancer cells with hBM-MSCs) were successfully produced and embedded into a matrix. This allowed for the observation of any changes in invasive patterns of both breast cancer cell types when cultured with hBM-MSCs (see Figure 2 and Figure 3) and vice versa, contributing to the fundamental investigation into the possible phenotypic and behavioural changes that may occur when hBM-MSCs and breast cancer cells interact. 3D culture of cancer cells provides the opportunity for cells to replicate the intimate cell–cell interactions that occur in vivo, creating a more accurate imitation of tissue structure, thus enhancing the clinical relevance of experimental results [26]. The ability of hMSCs to potentiate tumourigenesis and metastasis is well reported. hMSCs have been widely implicated in tumour support and are commonly found to home to tumour sites and secrete cytokines and matrix proteins that directly increase proliferation and metastases of cancer cells [27]. hMSCs also contribute to tumour growth via several other indirect mechanisms, including by transition to tumour-associated fibroblasts, suppression of the immune response, by promotion of angiogenesis, by stimulation of the EMT, through contribution to the tumour microenvironment, and by inhibition of tumour cell apoptosis [28]. 

However, studies also show that hMSCs can have anti-tumourigenic effects on cancer cells and can attenuate proliferation and metastases. Whilst our results show significant changes in the invasive activity of both hBM-MSCs and MDA-MB-231s when cultured together, co-culture with hBM-MSCs appeared to have an anti-metastatic effect on MDA-MB-231s when embedded into a matrix in vitro (see Figure 3 and Figure 4), and it is as yet undetermined whether there is a requirement for cell–cell interactions for these effects to be observed. Further studies are required to evaluate the nature of any interaction between the two different cell types, including proximity, and cell–matrix- and cell–cell-mediated signalling events. In addition, peripheral blood platelet lysate is a well-known alternative to FBS in the expansion and selection of MSCs in vitro [29]. It would be interesting to evaluate the impact of platelet lysate, not only on the invasive capacity of the hBM-MSCs used in the present study, but also on their anti-metastatic effects on MDA-MB-231 cells embedded in matrix.

The exact mechanism underlying possible anti-metastatic and tumourigenic effects of hMSCs is yet to be precisely defined. However, several studies suggest that the link lies in the effect of hMSCs on signalling pathways. For example, He et al. [30] demonstrated that hMSCs contribute to the reduced expression of mesenchymal markers such as vimentin and N-cadherin, and increased expression of E-cadherin, an epithelial marker, in MDA-MB-231 cells. They also showed that Stat3 activation, a pathway significantly related to tumour invasion and metastasis, was downregulated when MDA-MB-231 cells were cultured with hMSC conditioned medium. Similarly, it has been found that intravenously injected hMSCs delayed metastasis and decreased the frequency of lung metastases in mice [31]. One study found that MSC-derived extracellular vesicles (MSC-EVs) resulted in decreased cancer cell proliferation and more importantly, reduced cell migration [32]. They found that exposure to MSC-EVs lead to decreases in Ki67, a protein that is shown to correlate with metastasis. Additionally, it was found that when cultured with MSC-EVs for 24 h, cell adhesion potential increased; decreases in expression of the EMT marker vimentin and increases in expression of E-cadherin were observed. Existing literature provides some explanation behind the observed decreased invasion of breast cancer cells when co-cultured with hBM-MSCs in this study, supporting the results of this study. 

The development of this co-cultured 3D tumour-MSC spheroid model will allow further analysis of the impact on the invasive capacities of both cell types upon co-culture together. To expand this study beyond its current limitations of only utilising two breast cancer cell lines, more research needs to be conducted on additional cancer cell lines to determine whether there can be a generalisation of the anti-tumour effect of hBM-MSCs, and further molecular analyses may also be conducted to facilitate our understanding of the identified differences.

## 5. Conclusions

This study provides compelling evidence for the need for careful planning and decision making in future in vitro mammalian cell culture experimental conditions and provokes thought into the possible sources of variability that may inadvertently be integrated within experimental results. In this study, co-culture of hBM-MSCs and breast cancer cells under carefully defined co-culture conditions in 3D tumour spheroids allowed for the analysis of changes in invasive potential. Findings presented herein showed that co-culture of hBM-MSCs and MDA-MB-231 resulted in decreased invasion of both cell types. Previously, research on the role of hMSCs in tumour development and progression has mainly been conducted in 2D environments. Continuing to investigate the interaction between hMSCs and cancer cells in 3D systems is now essential to further understanding this relationship and the impact of tumour microenvironment, as well as increasing the possibilities of developing novel therapeutic techniques. Using 3D models such as the one used in this study provides an opportunity for in vitro relationships to be investigated and novel research on the mesenchymal stem cell-driven biochemical modulation of breast cancer cell metastases to be conducted.

## Figures and Tables

**Table 1:** Spectral characteristics of CellTracker™ (Thermo Fisher Scientific, UK) probes.

CellTracker™ Probe	Molecular Weight	Excitation (nm)	Emission (nm)
Green CMFDA	464.9	492	517
Orange CMRA	550.4	548	576
Deep Red	698.3	630	660

**Table 2:** Summary of analyses of growth parameters for each cell line in different growth media compositions. Displays mean values of growth parameters, standard deviation and significance of effects.

	hBM-MSCs					MDA-MB-231s					T47Ds				
Growth Media						GROWTH PARAMETERS									
	Day 7 (mean cells/cm^2^)	SGR (µ)	PDL	PDT (days)	FI	DAY 7 (mean cells/cm^2^)	SGR (µ)	PDL	PDT (days)	FI	DAY 7 (mean cells/cm^2^)	SGR (µ)	PDL	PDT (days)	FI
**LG-A**	16,867 * (±6861)	0.20 * (±0.10)	2.03 (±1.051)	2.88 (±1.45)	4.78 * (±3.145)	64,000 * (±14,751)	0.36* (±0.03)	3.65 * (±0.335)	1.05 * (±0.091)	12.8 * (±3.12)	43,200 (±13,685)	0.30 (±0.05)	2.99 (±0.48)	1.29 (±0.19)	8.25 (±2.91)
**HG-A**	13,333 (±7264)	0.17 (±0.10)	2.11 * (±0.89)	2.48 * (±1.035)	3.87 (±3.06)	52,800 (±14,221)	0.33 (±0.04)	3.36 (±0.41)	1.14 (±0.142)	10.56 (±2.96)	45,600 (±13,446)	0.30 * (±0.05)	3.02 * (±0.47)	1.28 * (±0.22)	8.40 * (±2.50)
**LG-B**	7800 (±3695)	0.10 (±0.03)	0.82 (±0.51)	4.98 (±0.31)	1.98 (±0.51)	47,000 (±8921)	0.32 (±0.03)	3.21 (±0.28)	1.19 (±0.10)	9.40 (±1.91)	23,680 (±2904)	0.22 (±0.01)	2.22 (±0.13)	1.72 (±0.09)	4.68 (±0.42)
**HG-B**	7466 (±2824)	0.11 (±0.11)	1.50 (±1.20)	12.55 (±17.70)	2.56 (±1.89)	38,400 (±6726)	0.29 (±0.02)	2.93 (±0.25)	1.31 (±0.11)	7.68 (±1.35)	30,480 (±4885)	0.22 (±0.08)	2.23 (±0.84)	1.58 (±0.30)	5.23 (±2.92)
	**OVERALL SIGNIFICANT EFFECT?**														
Serum	YES (*p* = 0.002)	NO ^#^	NO ^#^	NO ^#^	NO ^#^	YES (*p* = 0.004)	YES (*p* = 0.049)	YES (*p* = 0.048)	YES (*p* = 0.045)	NO ^#^	YES (*p* = 0.037)	YES (*p* = 0.036)	YES (*p* = 0.019)	YES (*p* = 0.042)	YES (*p* = 0.042)
Glucose	NO ^#^	NO ^#^	NO ^#^	NO ^#^	NO ^#^	YES (*p* = 0.005)	NO ^#^	NO ^#^	NO ^#^	NO ^#^	NO ^#^	NO ^#^	NO ^#^	NO ^#^	NO ^#^
Interaction	NO ^#^	NO ^#^	NO ^#^	NO ^#^	NO ^#^	NO ^#^	NO ^#^	NO ^#^	NO ^#^	NO ^#^	NO ^#^	NO ^#^	NO ^#^	NO ^#^	NO ^#^

* Indicates best performing condition. Colour coded green, yellow, orange and red to indicate best to poorest performance. # Denotes significance level of >0.05. **LG** = Low glucose, **HG** = High glucose. **Day 7** = Total cell number on day 7. **SGR** = Specific growth rate. **PDL** = Population doubling level. **PDT** = Population doubling time. **FI** = Fold increase.

**Table 3:** Chemical compositions of source A and B serums.

Chemical Test	Source A Serum	Source B Serum
Albumin	9 mg/mL	27.15 mg/mL
Cholesterol	31 mg/dL	127.4 mg/dL
Glucose	119 mg/dL	98.6 mg/dL
Haemoglobin	8.4 mg/dL	19.2 mg/dL
Immunoglobulin G	81 µg/mL	598 µg/mL
Osmolality	308 mOsm/kg	312 mOsm/kg
pH	7.20	7.28

## Supplementary Materials

The following are available online at https://www.mdpi.com/2306-5354/6/4/101/s1: Figure S1. Verification of MSC identity by analysis of MSC marker expression.

## Appendix A

Specific growth rate = µ = [ln(N_x_) − ln(N_0_)]/(t_x_−t_0_) [33,34,35].

Population Doubling Level = PDL= [log(N_x_) − log(N_0_)]/log(2) [36,37].

Population Doubling Time = PDT = ([log (2)]·[t_x_ − t_0_])/[log(N_x_) − log(N_0_)].

Fold increase = N_x_/N_0_ [35], where N_0_ and N_x_ are the cell numbers at the beginning and the end of the growth curve, respectively.

## Appendix B

Source A serum: Foetal Bovine Serum, Triple 0.1 µm filtered, US origin (FBS-BBT-5XM) supplied by Rocky Mountain Biologicals Inc. (Missoula, MT, USA).

Source B serum: FBS Good, EU approved (P40-37500), PAN Biotech, Wimborne, UK.
