# Supplementary material for: Determining Conditions for Successful Culture of Multi-Cellular 3D Tumour Spheroids to Investigate the Effect of Mesenchymal Stem Cells on Breast Cancer Cell Invasiveness
Source: Bioengineering (Basel). 2019 Nov 1;6(4):101. doi: 10.3390/bioengineering6040101 (PMC6955867; doi:10.3390/bioengineering6040101)
Supplement: Supplementary file 1 [file bioengineering-06-00101-s001.pdf]

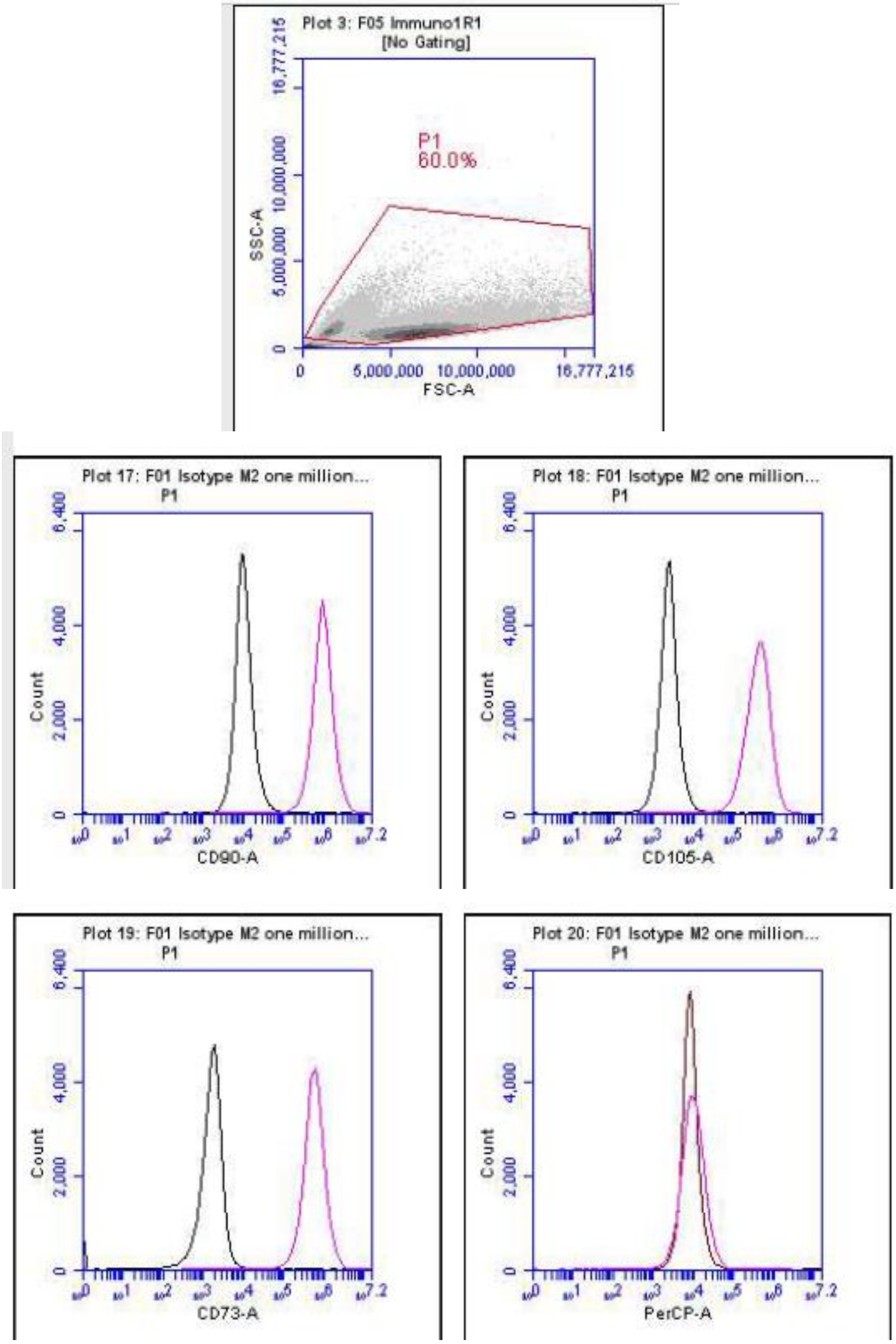

Figure S1: **Verification of MSC identity by analysis of MSC marker expression.** hBM-MSCs were stained for CD105 linked to PE, CD90 linked to FITC and CD73 linked to APC. Negative markers also stained for included CD14, CD20, CD34, CD45 and HLA-DR which were all linked to PerCP. The cells demonstrate the expected phenotype for hBM-MSCs (positive expression of CD105, CD90 and CD73, and negative expression of CD14, CD20, CD34, CD45 and HLA-DR.
